# The flow characteristics of solid particles used as additives for lubricants in the point contact area†

**DOI:** 10.1039/c7ra11313g

**Published:** 2018-03-06

**Authors:** Tianyi Sui, Baoyu Song, Feng Zhang, Yuanyuan Chen, Shuai Yan, Anying Wang, Mei Ding

**Affiliations:** School of Mechanical Engineering, Tianjin University Tianjin 300000 P. R. China suity@tju.edu.cn +86-22-27406951; School of Mechatronics Engineering, Harbin Institute of Technology Harbin Heilongjiang 150001 P. R. China

## Abstract

Solid nanoparticles have been applied as anti-wear and friction reduction additives for lubricants. In this paper, the flow characteristics of solid particles used as additives for lubricants were studied. A flow field visualizer based on high speed photography was developed. The particle trajectories in the point contact area were recorded using the flow field visualizer and were compared with the particle trajectories simulated using COMSOL. The results were analyzed and compared with tribological test results. It was found that the experimental results matched well with the simulation results, and the speed of the particles decreased by 60% at the inlet and outlet zones of the contact area. Solid particles were found to experience an unsteady flow in both the inlet and outlet zones and this would contribute to particle sedimentation in those areas, which matches well with the findings of the tribology test, which showed that more particles sedimented in those two areas.

## Abstract 2

The flow characteristics of solid particles used as lubricant additives under point contact conditions were investigated.

## Graphical abstract

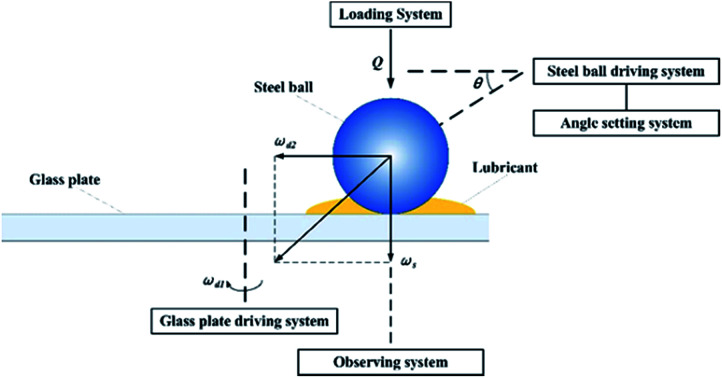

## 1. Introduction

Nowadays solid particles are added into lubricants for use as additives to improve the tribological properties, and the tribological performance of solid particles has been broadly investigated.^1–5^ These solid particles exhibit good anti-wear and friction reduction properties by adsorbing on the wear surface, forming a protective film, supporting the contact surface and repairing the rough surface.^6–9^ As one of the most important and interesting topics in the study of particle tribological mechanisms, the motion of particles in the contact area, especially for point contact and line contact conditions, has been the focus for many researchers.^10,11^ The motion of particle additives influences the functioning of the particles and the tribological properties. The surface would be damaged by the particles if a large number of particles became entrapped in the contacting area, especially under point contact conditions.^12,13^ Therefore, it is important to study the flow characteristics of particles for use as lubricant additives.

Among the different studies, there has been considerable interest in the motion characteristics of particles.^13,14^ Nikas carried out a mathematical study on the motion of small spherical solid particles in the inlet zone of elastohydrodynamic point contacts, and it was found that the particles may become entrapped in the inlet zone and particle rejection is associated with the risk of inlet blockage and fluid starvation, which may further cause film collapse and scuffing.^15,16^ Peymani simulated phase particle entrapment using ANSYS FLUENT and found that the particle size and the pressure–velocity boundary condition would influence particle entrapment dramatically.^17^ Benedetti studied the sliding motion of angular particles down a smooth plane and found that the gravity force of the particles was balanced by a self-tuned friction force and the downward velocity depended on both the contact angle and vibration of the plate.^18^ Besides simulation methods, particle image velocimetry (PIV) is another effective way to study the motion of particles. Sui studied the point contact flow field using a high speed camera.^19^ Haider studied the flow field of in-cylinder swirling flow using a particle image velocimetry technique.^20^

Keeping in mind the previous work reviewed above, particle additives could improve the tribological properties of lubricants and the study of the fluid dynamics of particles is important for understanding the mechanism of action of particle additives. However, the motion of particles in the contact area is hard to observe and limited research has focused on the visualization of the movement of particles in the contact area. Also, most researchers have focused on particle motion in the inlet zone and the contact zone, and few researchers have studied particle motion in the outlet zone, however particles would also easily become entrapped or sediment in the outlet area. It is necessary to give a whole view of the flow field in the contact area and to investigate particle motion by using both simulation methods and visualization methods.

In this paper, the motion characteristics of particles in the contact area were studied. In order to observe the flow field in the point contact area, a visualizer for the point contact flow field has been developed based on a high speed camera. The trajectories of the particles were observed using the flow field visualizer and the velocity field of the particles in the contact area was recorded. The results were compared with the simulation results, obtained using COMSOL. The experimental results matched well with the simulation results. The flow in the contact area is a laminar flow, while two vortex pairs were found near the inlet and outlet zones. The solid particles were found to experience complex flow in the outlet and inlet zones of the contact area. Some particles sedimented in the outlet zone as a result of the unsteady flow. The particle speed decreased by about 60% in the inlet and outlet zones, which could contribute to particle sedimentation. From the tribology tests, it was found that the particles aggregated and sedimented in the inlet and outlet zones on the wear surface, which matched well with the findings.

## 2. Experimental section

### 2.1 Particle trajectory simulation

A 2D model for the point contact area was set up in Auto CAD and imported into COMSOL. Based on Hertz contact theory, a ball will deform due to high pressure. So, the 2D point contact area was simplified as a ball with a platform on a flat surface. The top wall geometry of the contacting area was calculated and developed based on Hertz contact theory. The gap between the top and bottom surfaces was set as 20 μm and the ball diameter was set at 6.7 mm. The viscosity of the fluid was set at 0.5 Pa s and the density was set at 850 kg m^−3^. The velocity of the fluid at the inlet was 0 and the fluid was driven by the top and bottom walls, which represent the ball and plate. The sliding speed of the top and bottom walls was set at 0.5 m s^−1^. The particle density was set at 2200 kg m^−3^ and the diameter was set at 4 μm. In the simulation, the flow was set as a Newtonian fluid and the simulation was governed by the Navier–Stokes equation. The obtained mesh had 21 201 elements, as shown in Fig. S1.† The stationary flow field of the 2D point contact area was first calculated (shown in Fig. S2†), and then tracing particles were added into the fluid and were driven by the flow following the Stoke equation; the interaction between the particles and the fluid was neglected because of the limited load of the particles. The trajectories of the particles were recorded when flowing though the contact area.

### 2.2 Flow field observation of the solid particle

**Fig. 1:**
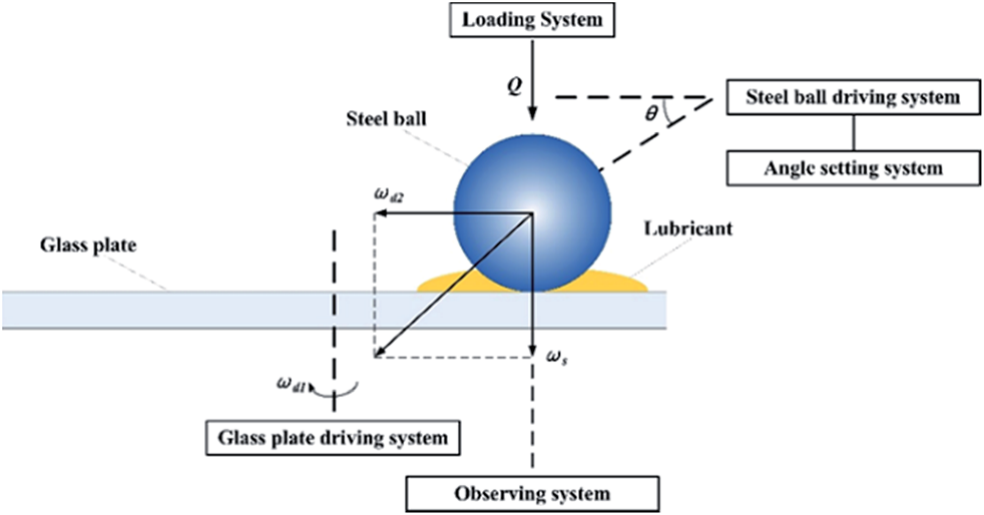
A schematic diagram of the flow field visualizer.

To simulate the working conditions of the angular contact ball bearing and to observe the point contact area, a test bench was designed as two systems, *i.e.* an image acquisition system and a point contact simulation system. As shown in Fig. 1, the point contact simulation system could be set at different speeds, loads and angles, and therefore it could be used to simulate the contact condition of the bearing ball in the angular ball bearing. At the same time, the contact area was magnified using magnification devices and captured using a high-speed camera. Data collected from the devices could then be transferred to a computer and analysed. A detailed drawing of the assembly of the flow field visualizer is shown in Fig. S3.† To simulate the real point contact conditions in the ball bearing (a steel ball rotating and sliding at the same time when working), both the steel ball and the plate were driven by motors separately. The steel ball driven by a servo motor was placed on the glass plate driven by a plate servo motor. The rotation speed could be controlled precisely from 0 to 3000 rpm. The contact angle could be tuned using the angle adjustment system from 30° to 60°. A zoom lens obtained from Navitar, Inc. with a magnification factor from 3.5 to 40 was mounted on a high-speed camera. The shooting speed was up to 153 846 frames per second.

To record the particle trajectories in the lubricant, silica particles were added into PAO as tracing particles. To ensure that the particle could flow though the gap in the contact area, the film thickness was calculated first. The minimum film thickness, calculated using the Hamrock–Dowson film thickness formula, was 11.8 μm and the central film thickness was 13.25 μm, and so the tracing particles could enter and flow through the contact area successfully. Silica particles with a diameter of 4 μm were added into a PAO 100 lubricant, obtained from Shanghai Fox Chemical Technology, and were mixed with rapid stirring. The lubricant was used as-prepared to avoid aggregation and sedimentation of the particles. The load was set at 70 N and the sliding speed was set at 500 mm s^−1^. The settings for the observation system are shown in Table 1.

**Table 1:** The parameters for the observation system

Parameter	Photo frequency (FPS)	Resolution ratio	Illumination time (μs)	Magnification factor	Imaging unit (μm)
Value	6420	480 × 480	153	3.5	11.5

### 2.3 The tribological experiment for solid particle additives in PAO lubricant

To determine solid particle motion under real application conditions, we dispersed silica particles in a PAO lubricant and tested the tribological performance. The preparation process for the silica particles and the lubricant was described in another paper published previously.^3^ The SEM images of the particles used in the experiment are shown in Fig. S4.† The tribological properties of the solid particles used as lubricant additives were examined using a MRS-10 four-ball tribometer. The four-ball test is a typical test method in which one steel ball under a load rotates against three steel balls that are held stationary, immersed in a lubricant. The detailed test procedure is listed in the ESI (Fig. S5†). The 12.7 mm diameter GCr15 steel bearing ball (grade 4) was obtained from the Shanghai Steel ball factory and cleaned ultrasonically with ethanol before the test. All tests were performed at room temperature under a load of 390 N, and a rotation speed of 1450 rpm for 30 min. The tribological tests performed for different lubricants were repeated three times under the same conditions. The coefficient of friction (COF) was recorded during the test and the average COF was calculated. The wear surface of the steel ball was examined using SEM and energy dispersive spectroscopy (EDS) analysis. The wear scar diameter (WSD) was also measured. After the tribology test the steel ball was put into an ultrasonic cleaner and cleaned for 15 min before characterization.

## 3. Result and discussion

**Fig. 2:**
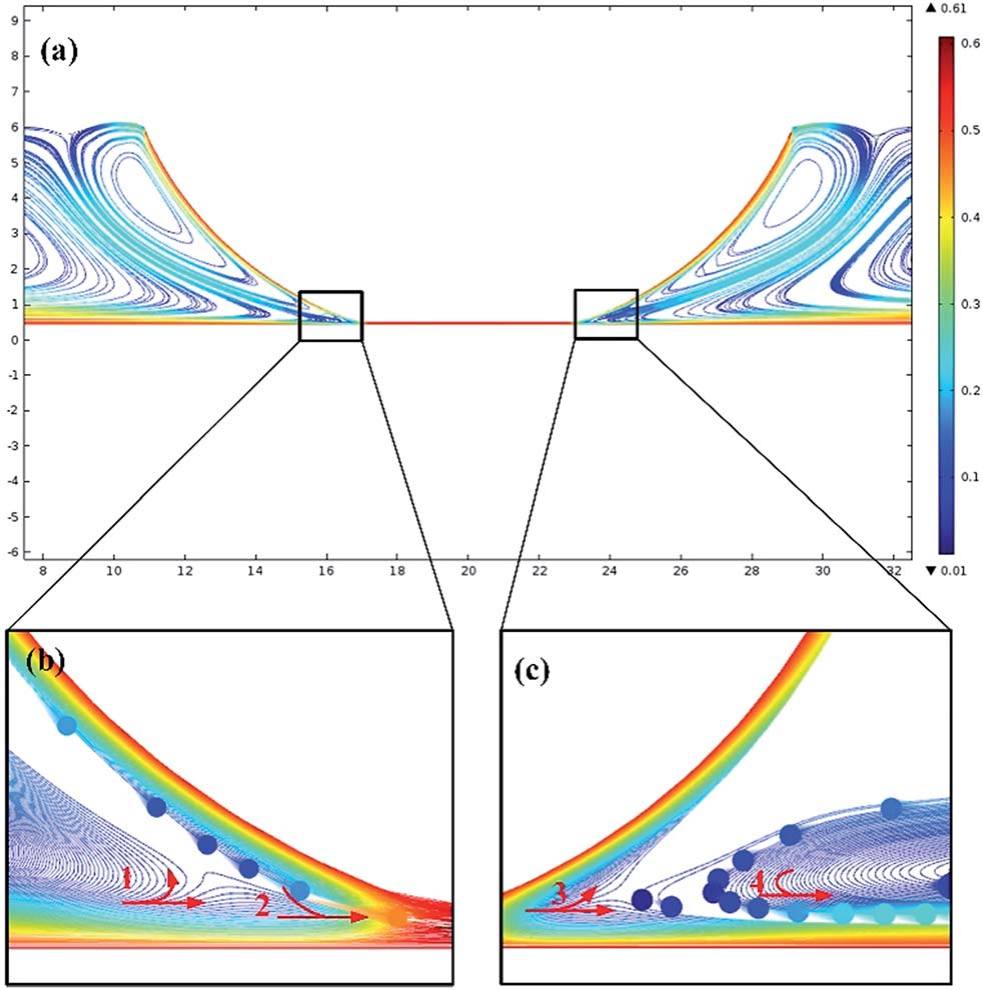
Simulation of the particle trajectories: (a) an overall view of the particle trajectories in the point contact area, (b) the particle trajectories in the inlet zone and (c) the particle trajectories in the outlet zone.

The motion of particles used as additives in lubricants was simulated using COMSOL. As shown in Fig. S2,† a 2D geometry for simulating the point contact conditions was set up. Both the top and bottom walls were set to slide in the same direction. According to Hertz contact theory, the geometry of the point contact will be deformed due to ultra-high pressure. Particles were added into the fluid and were dragged by the flow; the simulation results of the particle trajectories are shown in Fig. 2. The color bar represents the velocity of the particles and the units are m s^−1^. Fig. 2(a) shows the overall view of the particle velocity field. Particles near the sliding wall were dragged by the high speed flow and the speed of the particles was about 0.5 m s^−1^ while the speed of the particles far from the wall was below 0.2 m s^−1^. Two pairs of counter-rotating vortexes were found around the inlet and outlet zones. The particles in both the inlet and outlet zones were divided into two separated regions by the vorticity concentrated between the top and bottom walls. The flow inside the contacting area was a laminar flow and the flow trajectories of the particles inside the contacting area are shown in the ESI Fig. S6.†

Fig. 2(b) shows the particle trajectories in the inlet zone. In the inlet zone, as can be seen from red arrow 1, when the particles flowed towards the contact area, some of them were dragged by the fast sliding of the bottom wall and flowed into the contact area while other particles flowed backwards and flowed upwards. The particles then divided into two flows and flowed with the counter-rotating vortices separately. The particles that flowed with the vortex near the top wall would then be dragged by the top wall and would flow into the contact area. It can be seen from red arrow 2, that the particles that flowed with the top wall joined the particles that flowed with the bottom wall. The speed of the particles in the inlet zone increased substantially when they flowed into the contact zone. However, it needs to be noted that the separation of particle flow, shown by arrow 1, and the convergence of flow, shown by arrow 2, near the inlet zone would cause a drop in speed and a collision of the particles, and thus the solid particles would easily become entrapped in that area. This interpretation matches well with the results obtained by Professor Spikes.^21^

Fig. 2(c) shows the particle trajectories in the outlet zone. As shown by arrow 3, when the particles flowed out of the contact zone, some of them flowed away with the bottom wall and other particles were driven by the vortex and flowed upwards with the top wall and then flowed back to the bottom surface. As can be seen from arrow 4, those particles would join the flow near the bottom wall and the particle speed would increase from 0.1 m s^−1^ to 0.3 m s^−1^. It should be noted that the speed of the particles experienced a dramatic drop near the outlet zone. When the particles flowed back to the bottom surface, the particle speed would be lower than 0.1 m s^−1^ and the particle speed was hard to increase, which could be attributed to the counter-rotating vortices. The particles would easily sediment at low speed and a large area of deposited film may be formed in this low speed area of particles. The particle trajectories changed with time and are shown in the ESI Fig. S7.†

**Fig. 3:**
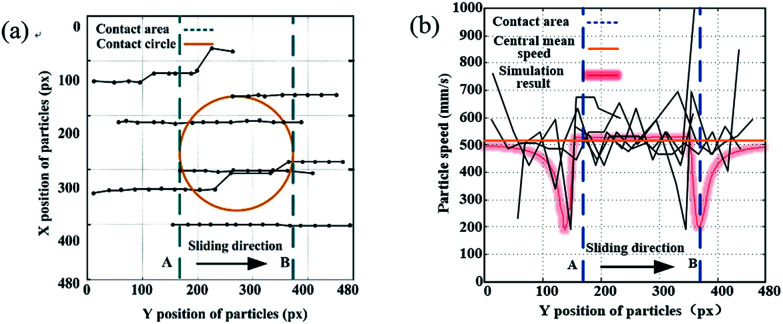
Velocity field of the particles in the contact area: (a) the *X* and *Y* positions of the tracing particles and (b) the velocity field of the tracing particles.

The flow characteristics of the particles used as additives in fluids were further studied using a flow field visualizer. Tracing particles with a size of 4 μm were added into the lubricant and the flow field was captured using a high-speed camera. The velocity field of the tracing particles is shown in Fig. 3. The circle in Fig. 3(a) represents the contact area circle. The blue dashed line A, shown in Fig. 3, represents the inlet zone and the blue dashed line B represents the outlet zone. It can be observed that most of the trajectories of the particles are parallel to the sliding direction. No intersection of the trajectories of the particles was found in the test, which indicates that the flow in the contact area was a laminar flow. However, when we look at the particle trajectories near the inlet zone, we can observe that both the particle velocity and the particle trajectories changed dramatically, which indicates the complex flow in this zone.

The speed of the particles was calculated based on the particle trajectories and the results are shown in Fig. 3(b). It can be seen from the figure that the speed of the particles fluctuated dramatically in both the inlet and outlet zones while they remained steady in the center of the contact area. Some particles slowed down in the inlet zone and the outlet zone, which could be attributed to the vortex in these areas. The red line shown in Fig. 3(b) represents the simulation results for the particle speed. It can be seen that the simulation results match well with the test results, in which the speed of the particles drops from approximately 0.5 m s^−1^ to about 0.2 m s^−1^ in the inlet zone, then rises to 0.5 m s^−1^ and remains stable through the contact area, and then drops down quickly to 0.2 m s^−1^ in the outlet zone.

The percentage of particles entering the contact area was calculated from both simulation and test results. Based on the test results, about half of the particles flowed through the contact area while the other half flowed away from the contact area. Based on the simulation results, about one fifth of particles flowed into the contact area with the bottom wall first, then another one fifth of particles flowed into the contact area with the top wall, and three fifths of particles flowed away from the contact area, driven by the vortex near the inlet zone.

**Fig. 4:**
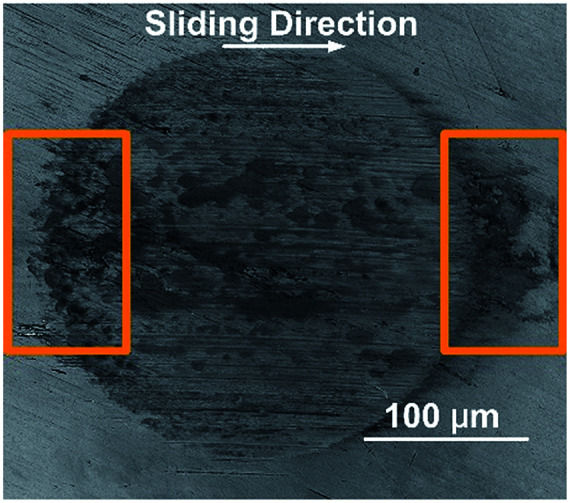
An SEM micrograph of the steel wear surface of PAO with functionalized silica nanoparticles.

Fig. 4 shows the SEM micrograph of the steel ball wear surface of PAO (poly-alpha olefin) with silica nanoparticle additives. The tribological properties of amino-functionalized silica nanoparticles in PAO were tested using a four-ball tribometer. The silica particle additives were proven to have good anti-wear and friction reduction properties, due to forming protecting films and filling into grooves, in our previous research.^3,9^ However, inhomogeneous particle adsorption was found on the wear scar. It can be observed from Fig. 4 that a dark grey area formed in the inlet zone. At the same time, a sedimentation area was found in the outlet zone, which looks like a comet tail of the wear surface. The roughness of the steel ball after the tribological test was measured using a laser scanning confocal microscope (LSCM), based on Standard ASME B46.1-2009. The surface average roughness (SRa) and the surface root mean square roughness SRq of the wear scar surface were 0.173 μm and 0.228 μm, respectively. In order to know whether the dark area was composed of steel wear debris or particle additives, a steel ball lubricated with pure PAO was tested for comparison and examined using SEM; the SEM micrograph is shown in Fig. S8 in the ESI.† The diameter of the wear surface increased dramatically, and deep furrows were found on the surface. However, no obvious dark area was found in the inlet zone and the outlet zone of the wear surface.

**Fig. 5:**
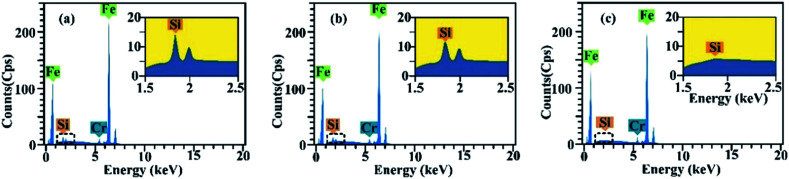
The EDS spectra of the wear surface: (a) the dark area on the wear surface of the lubricant with silica particles, (b) the light area on the wear surface of the lubricant with silica particles and (c) the wear surface of the lubricant without particles.

**Fig. 6:**
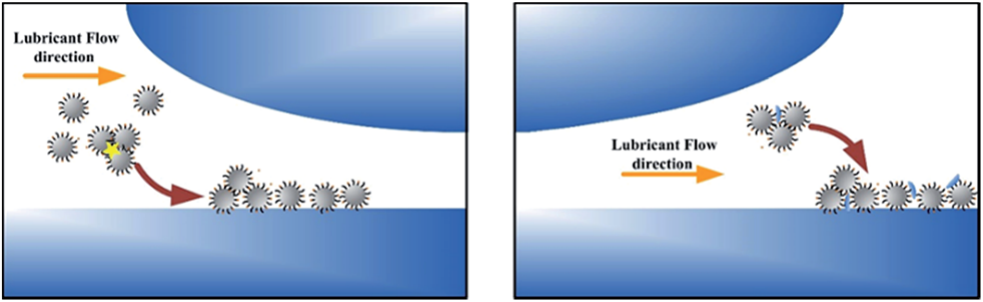
The mechanism of particle sedimentation in the outlet zone.

The wear surfaces of both pure PAO and PAO with particle additives were examined using EDS (shown in Fig. 5), and it was found that, besides the large peaks corresponding to Fe and Cr, the peak corresponding to Si was found around 2 keV. The dark area observed on the wear surface was found to contain larger Si peaks than the light colour areas, which suggests that a high density of silica particles sedimented in that area and formed a protective layer. For the wear surface with pure PAO, no Si peak was found in the spectrum of the wear scar surface. The EDS test shows a larger peak of Si in both the inlet and outlet zones when compared to that for other areas on the wear surface, which indicates that the particles are entrapped or adsorbed in those areas. This inference matches well with the findings from the flow field simulation and the observations, where in the inlet and outlet zones, the particle speed would decrease sharply and thus the particles would easily sediment or become entrapped in those areas. The mechanism of particle adsorption and sedimentation in the inlet and outlet zones is shown in Fig. 6. From the flow field simulation and observations, we know that in the EHD contact, the flow is divided into two regions in the inlet zone. One is the region where the flow can pass through the contact and the other is the reverse-flow originating from two counter-rotating vortices that reject part of the flow from the contact. When particles are taken into the inlet zone, the complex flow would cause particle collision and entrapment, also the speed of the particles would be slow in the inlet area, and so particles would easily sediment in those areas. When particles flow though the outlet area, the particles would experience a dramatic drop in their speed and some of the particles would remain at low speed in this region, which would cause aggregation of the particles and sedimentation, finally forming a comet tail area near the outlet region.

## 4. Conclusion

A flow field visualizer for observing point contact was developed based on a high speed camera. The flow characteristics of solid particles used as lubricant additives under point contact conditions were investigated. The flow field of the point contact conditions was studied using COMSOL software and a flow field visualizer. It was found that the flow in the contact region was mainly a laminar flow while a vortex pair appeared near the inlet and outlet zones. In the inlet zone, some particles flowed into the contact area while other particles flowed back with the top wall, and in the outlet zone, some particles flowed away with the bottom wall and other particles flowed with the top wall then flowed back to the bottom surface. By using the point contact flow field visualizer, solid particles were found to experience unsteady flow in both the inlet and outlet zones of the contact area. Both the simulation results and the observation results showed that the particle speed dropped by nearly 60% at the inlet and outlet regions, which would lead to particle sedimentation. This finding matches well with the tribology test of PAO with silica nanoparticle additives. About half of the particles entered the contact area while the other particles flowed away from the contact area, as can be seen from the simulation and test results. The particles were found to have aggregated and adsorbed in the inlet zone, forming a protective film, and had sedimented in the outlet zone of the steel ball wear surface, forming a comet tail region.

## Conflicts of interest

There are no conflicts to declare.

## Supplementary Material

RA-008-C7RA11313G-s001
