# Supplementary material for: The flow characteristics of solid particles used as additives for lubricants in the point contact area
Source: RSC Adv. 2018 Mar 6;8(17):9457–61. doi: 10.1039/c7ra11313g (PMC9078684; doi:10.1039/c7ra11313g)
Supplement: RA-008-C7RA11313G-s001 [file RA-008-C7RA11313G-s001.pdf]

Electronic Supplementary Material (ESI) for RSC Advances.
This journal is © The Royal Society of Chemistry 2018

The flow characteristic of solid particles as additives for lubricant in point contact area

Supporting Information:

1. Meshing of the geometrical model

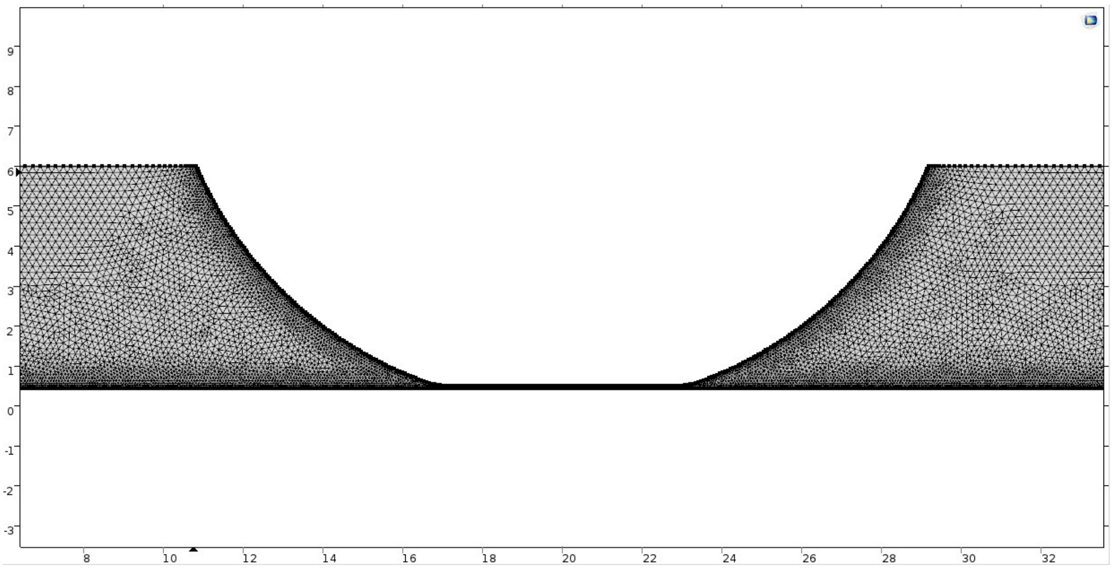

Fig. S1 Hexametrical meshing of the geometrical model

2. The flow field of 2D point contact area

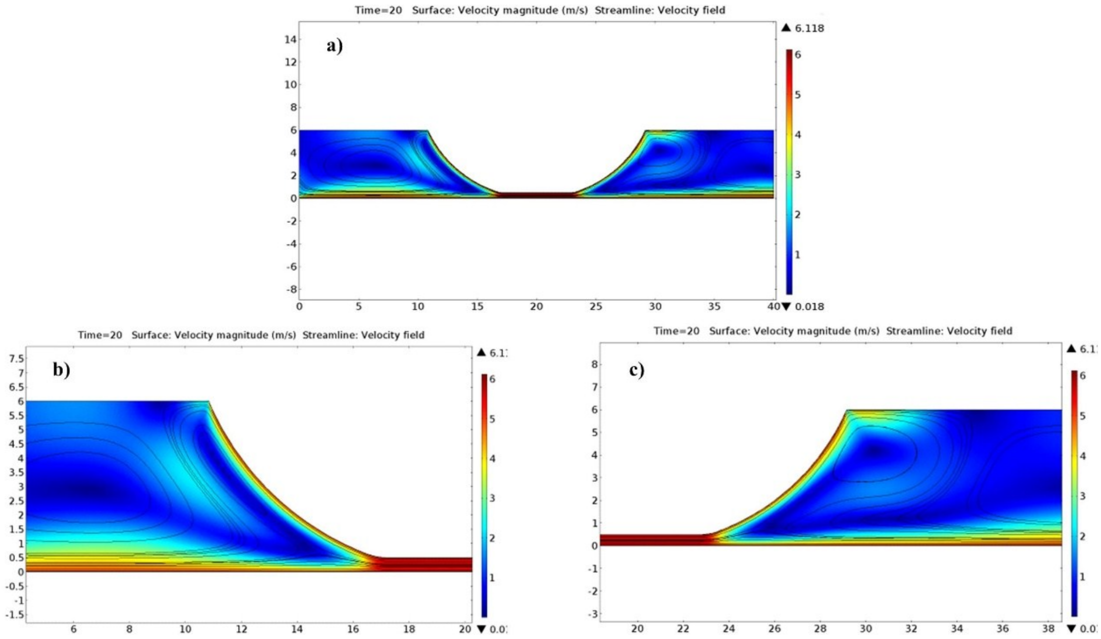

Fig. S2 The 2D flow field of point contact area: a) the whole picture of the flow field, b) the inlet zone of the flow field, c) the outlet zone of the flow field

3. The assembly drawing of the flow field visualizer

The assembly drawing of the flow field visualizer was shown in Fig. S3.

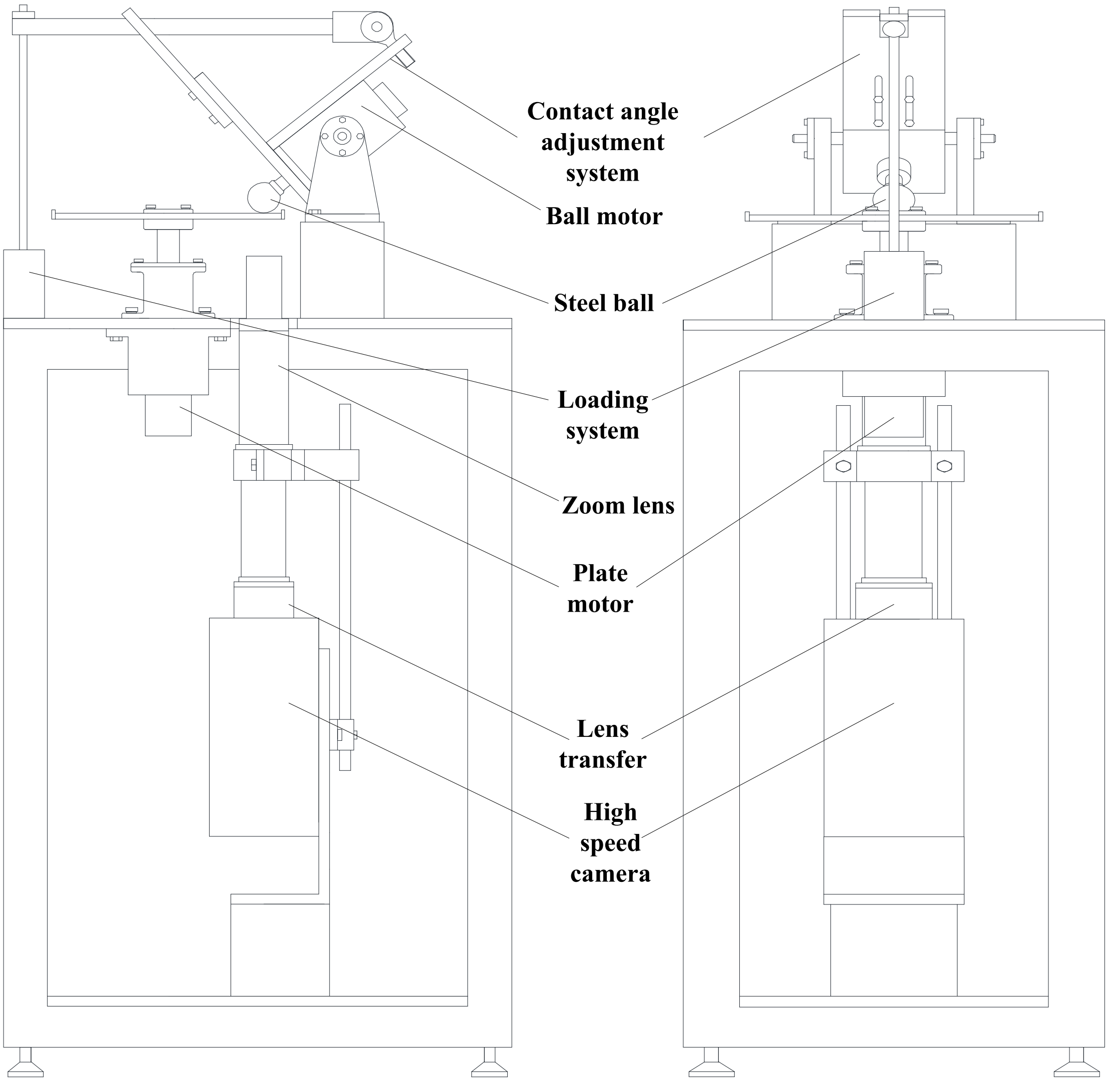

Fig. S3 The assembly drawing of the flow field visualizer

4. The SEM of the particle added into the PAO lubricant

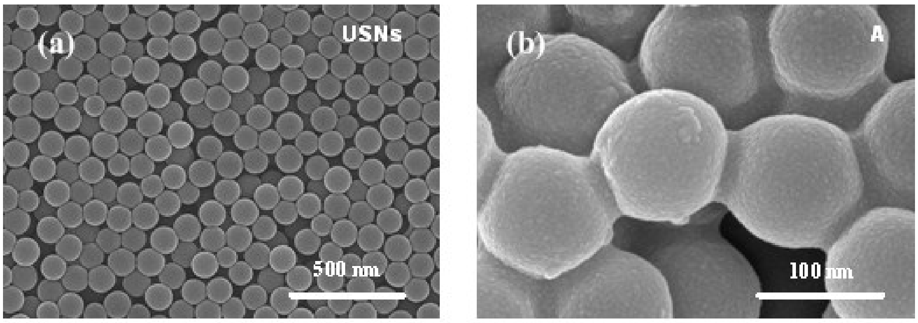

Fig. S4 The particle added into PAO lubricant: (a) The unmodified silica particles, (b) silica particles modified by amino functional groups.

5. Four-ball test method

Test procedure was list as following:

Place the three test balls in the test-lubricant cup. Place the lock ring over the test balls and screw down the nut securely. Pour the lubricating fluid to be tested over the three test balls until they are covered. Press one ball into the ball chuck and mount the chuck into the chuck-holder Install the test-lubricant cup assembly on the test apparatus in contact with the fourth ball. Place the spacer between cup and thrust bearing. Pladce the weight tray and sufficient weights on the horizontal arm in the correct notch for a base test load. Release the lever arm and gently apply the test load to the balls, making certain the cup assembly and spacer are centered. If the optional friction-measuring device is used, connect the calibrated arm on the test-lubricant cup to the indicator spring by means of the clip and wire.

The section view of four-ball tribometer was shown in Fig. S5

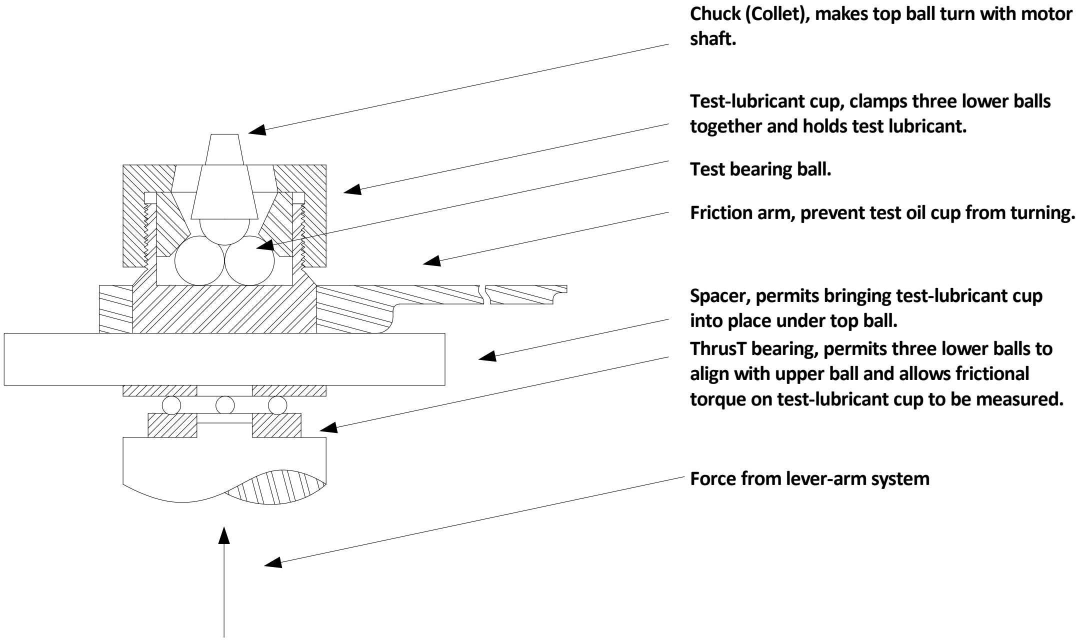

Fig. S5 Section View of Four-Ball Tribometer

6. The particle trajectories inside the contacting area

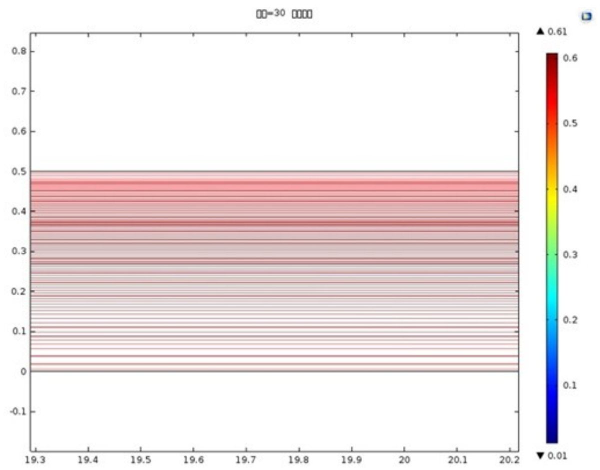

Fig. S6 The simulation result inside contacting area

7. The flow trajectory of particles in point contact area

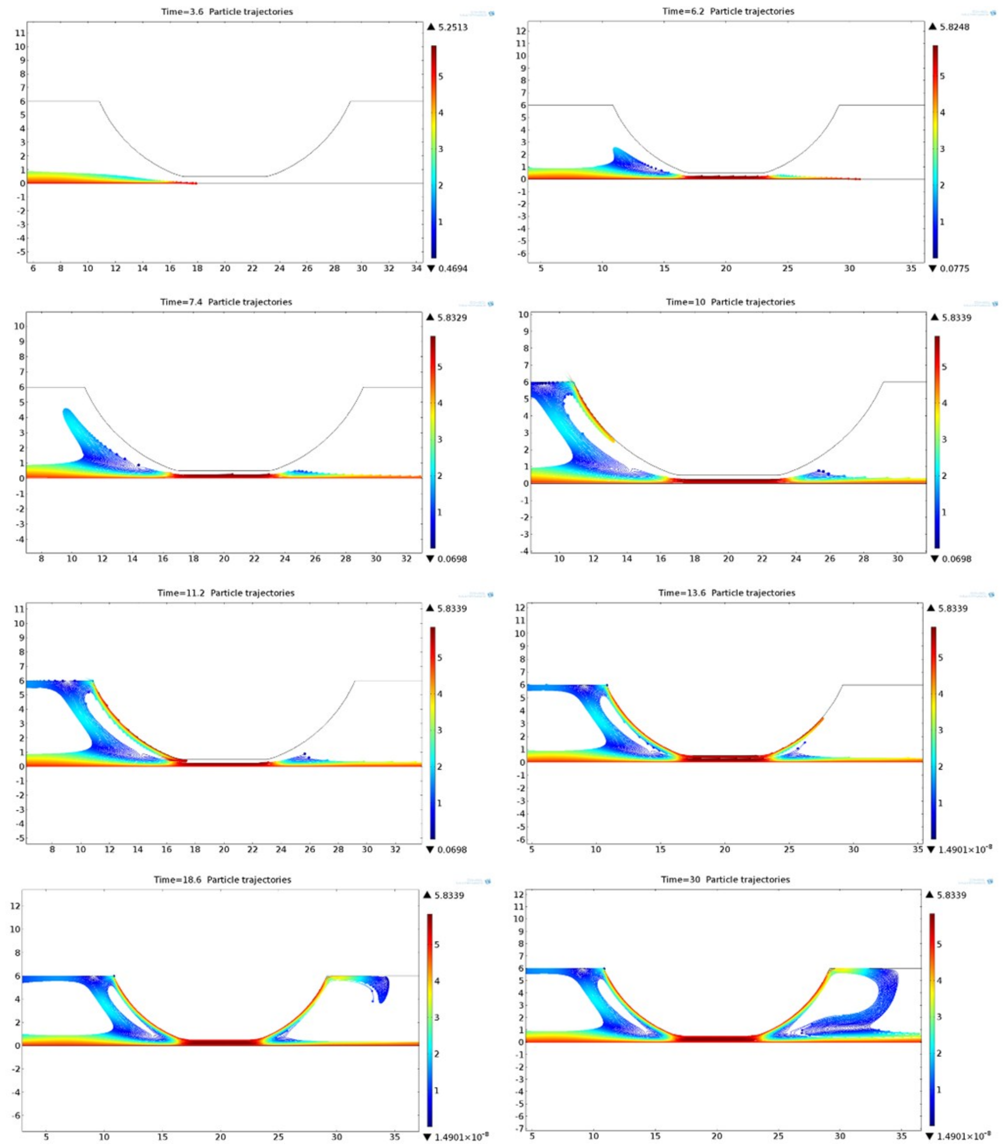

Fig. S7. The flow trajectory of tracing particles

8. The steel wear surface lubricated with pure PAO lubricant

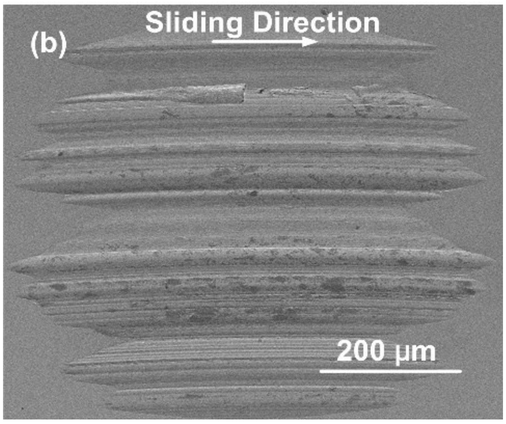

Fig. S8 The wear surface of steel ball lubricated with pure PAO lubricant
